# Mothers Do Not Show Increased Offspring Avoidance and Elevated Corticosterone Levels during Weaning Conflict in Rats

**DOI:** 10.1371/journal.pone.0163195

**Published:** 2016-09-23

**Authors:** Charlotte Cox, Reinmar Hager

**Affiliations:** Computational and Evolutionary Biology, Faculty of Life Sciences, University of Manchester, Manchester, M13 9PT, United Kingdom; Universite de Rennes 1, FRANCE

## Abstract

Parent-offspring conflict is predicted to occur because offspring will demand more parental investment than is optimal for the parent, and is said to be strongest during weaning when parents reduce nursing while offspring continue to demand parental care. While weaning conflict has been shown to be stressful in offspring, little is known about the effects of weaning conflict on mothers. We hypothesized that during weaning mothers have higher levels of stress hormone (corticosterone) compared to early lactation because of increased offspring demand. Further, we predicted that if mothers are given the option to avoid offspring solicitation they would do so and show lower corticosterone levels. We tested our hypotheses in an experimental population of rats in which one group of females was given the opportunity to avoid offspring solicitation. We measured faecal corticosterone metabolite levels using a non-invasive approach, and maternal and offspring behaviours during weaning. In contrast to our predictions, we detected lower levels of corticosterone metabolites during weaning than before, irrespective of cage type. Further, during weaning mothers did not show increased offspring avoidance behaviour although offspring solicitation increased significantly. Our results therefore cast doubt on the generally accepted notion of weaning conflict as a stressful period for mothers characterized by overt offspring solicitation.

## Introduction

Parent and offspring are predicted to be in an evolutionary conflict over the amount of parental investment, in particular during weaning. Based on Hamilton’s work in the 1960s, Trivers [1] developed the concept of parent-offspring conflict (POC) where offspring demand more than is optimal for the parent. The conflict arises because parent and offspring benefit from a given amount of investment to different degrees due to relatedness asymmetries. In monogamous species, a parent is related 50% to current and future offspring whereas offspring are completely related to themselves and thus value a given amount of investment twice as highly as the parent. Hence, parent and offspring have different optimal level of parental investment, the reason for the predicted conflict [1,2].

POC arises over any form of parental investment and thus stretches from early development until independence. Trivers referred to the weaning conflict as the most obvious manifestation of POC. During weaning parents are increasingly reluctant to respond to offspring solicitation while offspring continue to demand resources. Such weaning conflict has been observed in different taxa and is characterized by bleating and begging behaviours in both mammals and birds [3–6]. The degree of this conflict may depend on the ratio between maternal and offspring weight since providing resources is the greatest maternal cost. Rodents have a particularly high ratio of between 20–30% (cf humans have a ratio of 6; [7]) and are thus a highly suitable system to study the dynamics of POC. For example, König and Markl [8] have shown that females began to seek rest from day 17 postpartum as offspring attempted to initiate suckling more often. Weaning conflict is a stressful period for offspring [9]; however, little is known about whether the same applies to mothers. Given the elevated levels of offspring solicitation behaviours [1,8] weaning conflict may be associated with higher levels of maternal stress hormone and offspring avoidance behaviour.

In this study we investigated behavioural and hormonal traits during weaning conflict in two groups of rat mothers and their offspring comparing two cage environments, one of which allows females to escape from overt offspring solicitation. We predicted that during weaning corticosterone metabolite levels are higher during weaning then before, and that females who have the opportunity to escape their young have lower corticosterone levels than those that cannot.

## Material and Methods

### Experimental animals

We used outbred Sprague Dawley rats obtained from an in-house colony as one of the most commonly used models for behavioural phenotyping. To ensure experimental dams were familiar with their respective cage type we bred the F1 from four timed-mated females in their respective cage type. We determined sample sizes to balance power calculation estimates (using GPower 3.1.3) with welfare aspects of breeding large number of animals. Based on effect size estimates of 1.2 (Cohen’s d), an *α* level of 0.5 and power of 0.8, ten females were required in each group with their litters. F1 females were group-housed in their respective cage type until mating, weaned at 21 days of age, and were bred with an unrelated male from the same cage type at 65 days. Females were then housed individually in their respective cage types; weights of all females were recorded weekly and on days of behavioural observations.

Humidity ranged between 50% and 65% relative humidity, temperature between 20°C and 21°C. All animals were housed in a temperature-controlled environment on a 12-h light/dark cycle with the light period being from 07:00h to 19:00h. A fixed cage cleaning routine was implemented to ensure all animals experienced the same cage-change routine, and all animals were given ad lib access to food (Special Diets Services expanded pellets, Witham, UK) and water throughout.

### Experimental Conditions

Two laboratory cage types were used in this study (Figs A and B in S1 File): one type that allowed females to use an upper level that cannot easily reached by young rats, and one standard cage without this feature. The standard cage type had the following dimensions: L x W x H in mm: 580 x 380 x 190, floor area: 2204cm^2^. The two-level cage was individually ventilated with the following dimensions: L x W x H in mm: 462 x 403 x 404, floor area: 1800cm^2^. The upper level was L x W in mm: 410 x 250, floor area: 1025 cm^2^; thus totalling 2825 cm^2^ for the entire cage. All females were provided with Aspen wood chip bedding and sizzle nesting material. The day matings were set up was day 1 of gestation. To provide a baseline for female stress levels before parturition, faecal samples were collected on days 6, 10, 14 and 18 of gestation between 13:30 and 17:30 as soon as a sample was produced. In addition, faecal samples were collected from each female every other day of lactation, with the last samples collected on day 20. Samples were frozen and processed at the Endocrine Diagnostic Laboratory, Chester Zoo, following an established enzyme immunoassay (EIA) method to measure the amount of corticosterone metabolites in each sample [10]. Litter birth marked day 1 of lactation.

### Behavioural Observations

Behavioural observations were carried out during the light phase on postnatal days 10, 14, 16 and 18 to cover the period of the weaning conflict, and the period shortly before and after, following a pilot study. We standardised observational conditions to ensure that females and their litter were motivated to the same degree to show maternal and offspring interaction during experimental observations following Hager and Johnstone [4,11] and Lyst et al. [12]. Thus, mothers and their litters were separated for four hours before being reunited and observations commenced. Mothers were then placed in a new cage with the food and water from the original cage while the litter was left in the original cage and placed on a heat mat to keep the pups warm during the separation. On each of the four days, mothers and offspring were then observed for 15 minutes using a scan sampling technique [13], recording maternal suckling, activity, cage use as well as offspring solicitation, every 20s. Offspring solicitation behaviour was defined as pups attempting to suck and following the mother. Offspring behaviours were recorded for the entire litter. In the statistical analysis we then adjusted for litter size differences by adding litter size as a covariate to the model. All procedures were approved by the University of Manchester Ethics Committee, and did not require a personal license according to the UK’s Animals (Scientific Procedures) Act 1986. No discomfort or adverse effects were observed.

### Corticosterone measurements

All samples were analysed by an enzyme immunoassay using corticosterone antibody (CJM06). During gestation samples were collected by placing the females individually in a clean cage of their specific type with a metal grill base, thus allowing easy identification of which sample was produced by which female. Females were left on the metal grills for no longer than four hours, which was sufficient for the production of a sample. Urine contamination of faecal samples may dilute the concentration of corticosterone metabolites [14], as urine contains a different concentration of corticosterone metabolites [15]. Therefore to prevent any collection of contaminated samples, sugar paper was placed underneath the grill to make any urine passed by the female easily visible. After parturition, the freshest and uncontaminated faecal samples were taken straight from the home cage on observation days. All samples were collected between 13:30 and 17:30 in order to collect faeces from similar points of the circadian rhythm.

### Enzyme Immunoassay

All samples were analysed by an enzyme immunoassay (EIA) at Chester Zoo, UK. The assay involves competition between a known, labelled antigen and an unlabelled antigen in the samples, to bind to an antibody. The amount of resulting product produced by the binding of antigen and antibody can then be measured.

Firstly the corticosterone metabolites were extracted from the faeces in a two day process. On the first day of extraction samples were defrosted, and, once thawed, 0.5g of each faecal sample was measured out and placed into an extraction vial. Once all samples were placed in extraction vials, 0.5 ml of Milli-Q water and 4.5ml of methanol was added to each vial and vortexed for 10 seconds. All samples were placed on an orbital shaker and agitated overnight. On the second day of extraction, all vials were centrifuged for 20 minutes at 1800rpm. After centrifugation, the supernatant (methanol) was poured into test tubes and dried down in a warm water bath (~ 50°C), in a fume cupboard. Once all liquid was evaporated from the samples, they were re-suspended in 1ml of methanol. Test tubes were covered with parafilm to prevent evaporation before being vortexed and sonicated for 15 minutes to remove residue from the sides of the test tubes. After sonication, the test tubes were vortexed and the extract was poured off into plastic tubes and stored at -20°C.

### Biological Validation

Since this assay had not been used for rats before a biological validation was necessary to determine whether a CJM06 antibody used in the assay is able to bind and react with the appropriate hormone metabolites. The validation also acts to determine the correct dilution of the faecal extract with assay buffer when running a corticosterone assay for this species.

Samples were collected hourly from two female Sprague-Dawley rats of the same age (280- 300g), over a period of 24 hours in order to carry out the biological validation. For preparation of the samples, an equal amount (approximately 0.50g) of faeces extract from each sample, from each female, was pooled. The pool of neat faecal extract was diluted serially two-fold with 200uL of assay buffer in the following dilutions: neat, 1:2, 1:4, 1:8, 1;16, 1:32, 1:64, 1:128, 1:256, 1:512, 1:1024, 1:2048, 1:4096, 1:8192.

The percentage binding of samples was plotted by choosing an arbitrary concentration for the neat sample and halving the concentration for each dilution, if the sample curve plotted parallels the standard curve then the hormone in the sample being measured is immunologically similar to the standard and can be measured in the experimental species. The correct dilution to run samples at is shown on the curve at 50% binding, which was found to be 1:120 for samples from this particular species.

### Enzyme Immunoassay Protocol Antibody Preparation

Corticosterone antibody (CJM06) was diluted to 1:100 by adding 20uL to 2000uL of coating buffer. 100uL was then put into vials and frozen.

### Horseradish Peroxidase (HRP) Conjugate Preparation

Corticosterone-HRP was diluted to 1:100 by adding 25 uL of stock to 2.475 uL of EIA assay buffer and stored at 4°C.

### Standards Preparation

1mg of Corticosterone (Sigma Diagnostics; Cat #C2505) was added to 1 mL ethanol for a 1mg/mL primary stock solution. 1ml of primary stock (1 mg/mL) was added to 99 mL of ethanol assay buffer for a 1 mg/100mL (10,000 ng/ml) working stock in alcohol. This was diluted further (1:500) by adding 100 uL of working stock to 49.9 mL of EIA buffer to prepare 20ng/Ml, or 1000 pg/well of standard. All stocks were stored at -20°C.

The enzyme immunoassays were carried out in a three day process. On day one, 33.3uL of antibody stock (1:100, -20°C) was added to 5mL of coating buffer; the appropriate numbers of Nunc Maxisorp^™^ plates were coated by adding 50uL of this antibody solution in each well, apart from the first column as this was left as a blank. The plates were then covered with acetate plate sealer and left overnight at 4°C; the plates were ready to use by day three. On day three, the ready prepared standard stock was diluted serially 2-fold, using 200ul assay buffer, to the values: 1000, 500, 250, 125, 62.5, 31.2, 15.6, 7.8, 3.9pg corticosterone / well. The faecal extract from each of the samples was then diluted to the appropriate dilution with EIA buffer (1:120).

The procedure for running the plates began with adding 7.14uL of HRP conjugate stock to 5mL of EIA buffer. The plate washer was purged and all Nunc Maxisorp^™^ plates were washed five times with wash solution. 50uL of the diluted standard, samples and controls C1 and C2 was added per well. Immediately after this, 50uL of the diluted HRP Conjugate was added per well. The plates were then covered and left to incubate at room temperature for two hours in the dark. After the two hour incubation period the plates were washed five times with wash solution; plates were then left in the dark until dry. A substrate buffer was prepared immediately before use by combining at room temperature: 40 uL of H2O2, 125 uL of ABTS and 12.5 Ml of citrate buffer. 100uL of the substrate was then added to all wells of the Nunc Maxisorp^™^ plates, and the plates were covered and left to incubate at room temperature in the dark until the wells reached 1.0 optical density. The ABTS in the substrate buffer detects the kinetics of any hydrogen peroxide producing enzyme reaction between the antigen and antibody. The ABTS reacts with any hydrogen peroxide and produces a green, soluble end product. The plates were read by a spectrophotometer at 405nm; the light absorption of the end product was measured and therefore the amount of corticosterone metabolites in the samples could be calculated in ng/g of faeces.

### Statistical Analysis

IBM SPSS Statistics version 20 was used to run univariate and repeated-measures General Linear Models (GLMs). Repeated-measures GLMs were used in order to test data collected from the same subject across different days. In the models, categorical data such as cage type were defined as a fixed factor while continuous data, such as corticosterone metabolite level or body weight, were defined as a covariate, as specified for the individual models in the Results section. Significance testing was based on F-tests, with a significance level of *p* < 0.05.

## Results & Discussion

**Fig 1:**
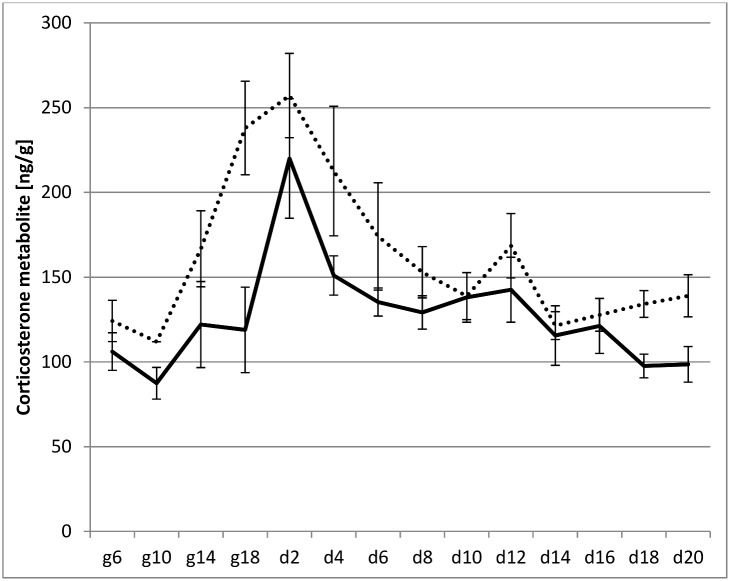
Mean corticosterone metabolite levels during gestation (gd) and lactation (d1-d20) until postpartum d20 for females in standard (solid line) and two-level cages (dotted line), with standard error bars.

Our key prediction was that weaning conflict is a period for mothers characterized by increased offspring avoidance behaviour and corticosterone levels compared to lactation prior to weaning. In rats, we defined the weaning conflict period as postpartum day 14–20 [8,16], which coincides with the increased ability of normally developed rats to feed independently on solid food. We begin testing our prediction by analysing predictors of corticosterone metabolite levels during gestation, early lactation and weaning. Fig 1 illustrates clearly that as gestation nears parturition, levels rise significantly and more than double around birth to up to 250 ng/g. Corticosterone metabolite levels then decreased continuously until day 10, and, from day 14 onwards, levels returned to a level similar to that of the baseline. Fig 1 shows corticosterone levels for our two groups of females: those who had the opportunity to escape their offspring by using an upper level in the cage the young cannot easily reach (‘two-level cage’; Fig B in S1 File), and those who did not have this opportunity (‘standard cage’; Fig A in S1 File). Our prediction was that the former group would exhibit lower levels of corticosterone than the latter because they can avoid excessive solicitation by their young during weaning. However, in contrast to our prediction, Fig 1 suggests that females in two-level cages had higher levels throughout, although the difference to females housed in standard cages is not statistically significant. We note here that the two-level cage had an overall larger size by about 20x30cm (the second level), which may affect behavioural patterns over and above effects of providing an opportunity to avoid offspring solicitation. Indeed, in a repeated measures GLM correcting for differences in female bodyweight, littersize and prebirth average corticosterone levels, no effect of cage type was found (GLM, *F*_*1*,*15*_ = 0.43, *p* = 0.52). When comparing average levels of corticosterone between pre-weaning and weaning (d14-20) it becomes evident that preweaning levels (d2-12) were significantly higher in both groups (two-level cage: T-test, *t(9)* = 3.34, *p* = 0.009. Standard cage: T-test, *t(9) =* 7.18, *p* <0.001). Thus, against our predictions, the period of weaning conflict is not associated with elevated stress levels in females, and females who were able to avoid offspring solicitation behaviour did not have lower stress levels than those who did not have this opportunity. While generally lactating rat mothers have been reported to show reduced anxiety (e.g. [17]), the presence or absence of offspring when exposed to a stressor has been shown to influence the degree to which lactating mothers show stress responses, with higher levels if the stressor might represent a threat to the litter (e.g. male intruder; [18]). Although in our study the removal of pups prior to behavioural recording may act as a stressor, and females in the two cage types may respond differently to this, we did not detect elevated corticosterone levels on days observations occurred compared to days without observations (cf days 10–16 vs before and after in Fig 1).

**Fig 2:**
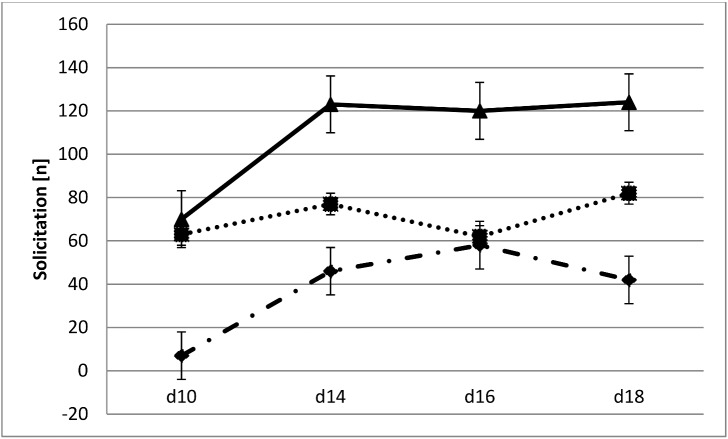
Offspring solicitation during weaning. The dotted line refers to the sum of solicitation behaviours shown by offspring in two-level cages. The solid line shows the sum of offspring solicitation in standard cages and the dashed line shows the sum of the two, with standard error bars.

**Fig 3:**
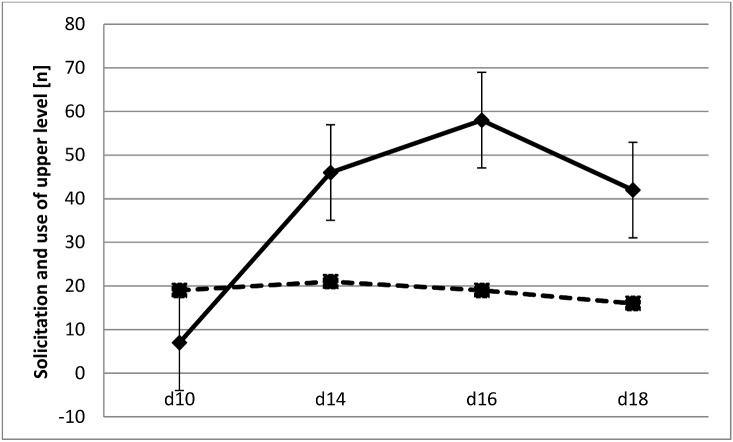
Solicitation and use of upper level by mothers during weaning with standard error bars. The solid line shows the sum of solicitation behaviours by offspring in two-level cages and the dashed line shows the use of the upper cage level by their respective mothers.

Given we predicted that mothers show increased corticosterone levels during weaning due to increased levels of offspring solicitation [8], the absence of increased levels could be due to offspring in our experiment not showing increased levels of solicitation behaviour. We have measured offspring solicitation and found overall an increase in offspring solicitation over the weaning period, but only in offspring in two-level cages (Repeated measures GLM, *F*_*3*,*7*_ = 16.14, *p* = 0.002; Fig 2). Overall, solicitation levels in standard cages were significantly higher than in two-level cages (repeated measures GLM, *F*_*1*,*18*_ = 10.767, *p* = 0.004). We further hypothesized that increased offspring solicitation would lead to increased use of the upper cage level by their mothers, which offspring even at postpartum d18 rarely reach. However, there was no effect of offspring solicitation on the frequency of the use of the upper level by mothers (Fig 3; Table A in S1 File). Now that we have established that offspring solicitation increases overall during weaning, we can ask whether mothers respond by increased suckling, given they are not avoiding pups. Surprisingly, the effect of solicitation on maternal suckling is significantly negative for all days, independent of cage type (e.g. d14, GLM, *F*_*1*,*17*_ = 7.05, *p* = 0.017; Table B in S1 File), again controlling for litter size effects. The more offspring solicit, the less mothers respond.

## Conclusion

We set out to test the hypothesis that the weaning period is characterized by higher levels of maternal corticosterone. Our results clearly find no support for this hypothesis. Although offspring solicitation increases during weaning, those mothers who have the opportunity to avoid being followed by offspring do not increase their use of a part of the cage offspring cannot easily reach. This is not because this upper level is particularly unattractive to mothers since the use of this cage part remained fairly constant, and mothers do not show increased corticosterone levels. The negative relationship between solicitation and maternal response (i.e. suckling) within families, during the weaning period clearly shows that mothers become less responsive overall the more offspring solicit. While in this study we only look at mothers and biological offspring, we found, using a mouse model, the same negative relationship in a cross-fostering design, which was likely due to increased cost of solicitation [19]. It is well established that during lactation the maternal brain changes in responsiveness to stressors [20], for example with increased glucocorticoid receptors in the hippocampus [21], and this may provide mechanisms by which changes to responsiveness to offspring solicitation are enabled. Further, maternal effects and conditions experienced during rearing may influence behavioural patterns seen at the adult level.

What do our results add to understanding POC? The theoretical foundation of the battleground is well established [1, 22] as is the behavioural manifestation of POC (squabbling, begging and similar) in mammals and other taxa (which may depend on whether the mother is pregnant again [23]). However, to demonstrate differential fitness consequences for parent and offspring, future work could manipulate resource allocation, which we predict would lead to a fitness increase in one and a decrease in the other party [24].

Bateson [25] pointed out that evolutionary conflict may not necessarily imply behavioural conflict. Indeed, the conflict may have been resolved evolutionarily and one may not expect to see signs of overt conflict. The absence of increased maternal avoidance behaviour and corticosterone levels in our study lends support to this view. Nevertheless, our study showed an increase in solicitation behaviour but mothers are not too troubled by this as evinced by the lack of increased use of the second cage level during weaning (offering the opportunity to avoid solicitation), and unchanged maternal corticosterone levels. Even if the latter are attenuated during weaning due to the high levels seen just before birth, the very small increase in corticosterone metabolite levels around the onset of weaning (d12, Fig 1) tails off rapidly and stays low until weaning is complete. We thus conclude that weaning is associated with increased offspring solicitation, but we found no evidence for increased maternal stress (as measured in differences in corticosterone levels) or offspring avoidance behaviour.

## Supporting Information

S1 File **Fig A in S1 File The standard cage used in the study.** The standard cage has one level and the following dimensions (L x W x H in mm: 580 x 380 x 190, floor area: 2204cm^2^). **Fig B in S1 File The two-level cage used in the study.** The two-level cage has the dimensions (L x W x H in mm: 462 x 403 x 404, floor area: 1800cm^2^) and includes a shelf with dimensions: L x W in mm: 410 x 250, floor area: 1025cm^2^ which provides a second level. A rat can be seen on the upper level looking down.**Table A in S1 File** General linear model, SPSS 22, showing statistics for effects of offspring solicitation behaviour and female activity on the use of the upper level by mothers, for each of the four days during weaning. **Table B in S1 File** General linear model, SPSS 22, showing statistics for effects of offspring solicitation behaviour and litter size (ls) on maternal suckling, for each of the four days during weaning.
(DOCX)
