# Supplementary material for: Mothers Do Not Show Increased Offspring Avoidance and Elevated Corticosterone Levels during Weaning Conflict in Rats
Source: PLoS One. 2016 Sep 23;11(9):e0163195. doi: 10.1371/journal.pone.0163195 (PMC5035065; doi:10.1371/journal.pone.0163195)
Supplement: S1 File — Fig A in S1 File The standard cage used in the study. The standard cage has one level and the following dimensions (L x W x H in mm: 580 x 380 x 190, floor area: 2204cm2). Fig B in S1 File The two-level cage used in the study. The two-level cage has the dimensions (L x W x H in mm: 462 x 403 x 404, floor area: 1800cm2) and includes a shelf with dimensions: L x W in mm: 410 x 250, floor area: 1025cm2 which provides a second level. A rat can be seen on the upper level looking down.Table A in S1 File General linear model, SPSS 22, showing statistics for effects of offspring solicitation behaviour and female activity on the use of the upper level by mothers, for each of the four days during weaning. Table B in S1 File General linear model, SPSS 22, showing statistics for effects of offspring solicitation behaviour and litter size (ls) on maternal suckling, for each of the four days during weaning. (DOCX) [file pone.0163195.s001.docx]

**Supplementary File to** PONE-D-16-22205

Mothers do not show increased offspring avoidance and elevated corticosterone levels during weaning conflict in rats by C. Cox & R. Hager

**Supplementary Figures A and B**


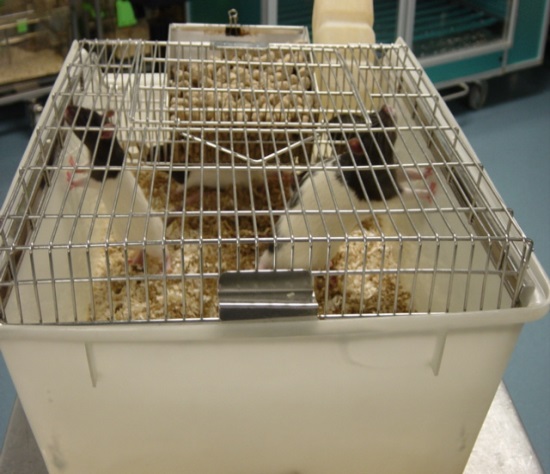


**Supplementary Fig A.** The standard cage used in the study. The standard cage has one level and the following dimensions (L x W x H in mm: 580 x 380 x 190, floor area: 2204cm²).


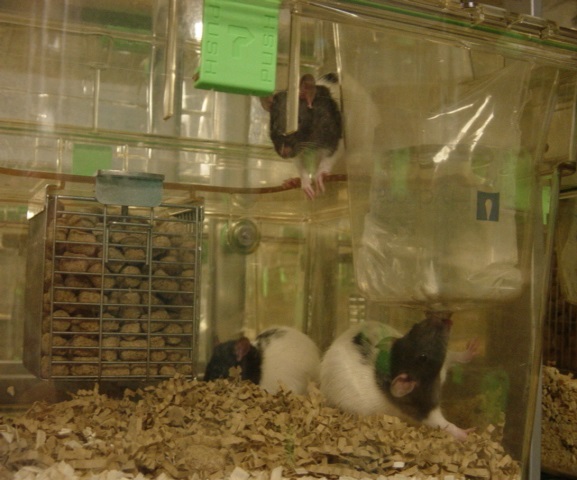


**Supplementary Fig B.** The two-level cage used in the study. The two-level cage has the dimensions (L x W x H in mm: 462 x 403 x 404, floor area: 1800cm²) and includes a shelf with dimensions: L x W in mm: 410 x 250, floor area: 1025cm² which provides a second level. A rat can be seen on the upper level looking down.

**Supplementary statistics results**

Table A

General linear model, SPSS 22, showing statistics for effects of offspring solicitation behaviour and female activity on the use of the upper level by mothers, for each of the four days during weaning.

| Day 12 solicitation | *p* = 0.532 | *F_1,7_* = 0.433 | female activity | *p* = 0.03 | *F_1,7_* = 19.254 |
| --- | --- | --- | --- | --- | --- |
| Day 14 solicitation | *p* = 0.832 | *F_1,7_* = 0.048 | female activity | *p* = 0.11 | *F_1,7_* = 3.385 |
| Day 16 solicitation | *p* = 0.620 | *F_1,7_* = 0.620 | female activity | *p* = 0.08 | *F_1,7_* = 4.202 |
| Day 18 solicitation | *p* = 0.073 | *F_1,7_* = 4.444 | female activity | *p* < 0.001 | *F_1,7_* = 41.176 |

Table B

General linear model, SPSS 22, showing statistics for effects of offspring solicitation behaviour and litter size (ls) on maternal suckling, for each of the four days during weaning.

| Day 12 solicitation | *p* = 0.006 | *F_1,17_* = 9.806 | Litter size | *p* = 0.278 | *F_1,17_* = 1.254 |
| --- | --- | --- | --- | --- | --- |
| Day 14 solicitation | *p* = 0.017 | *F_1,17_* = 7.051 | Litter size | *p* = 0.126 | *F_1,17_* = 2.589 |
| Day 16 solicitation | *p* = 0.049 | *F_1,17_* = 4.500 | Litter size | *p* = 0.360 | *F_1,17_* = 5.180 |
| Day 18 solicitation | *p* = 0.002 | *F_1,17_* = 13.108 | Litter size | *p* = 0.029 | *F_1,17_* = 5.704 |
